# Synthesis and biological activity of fused tetracyclic Pyrrolo[2,1-c][1,4]benzodiazepines

**DOI:** 10.1016/j.heliyon.2018.e00539

**Published:** 2018-03-01

**Authors:** Joel K. Annor-Gyamfi, John M. Jarrett, Joseph O. Osazee, Dobrusia Bialonska, Crystal Whitted, Victoria E. Palau, Abbas G. Shilabin

**Affiliations:** a Department of Chemistry, College of Arts and Sciences, East Tennessee State University, TN, 37614, USA; b Department of Biology, University of North Georgia, 82 College Cir, Dahlonega, GA, 30597, USA; c Department of Pharmaceutical Sciences, Gatton College of Pharmacy, ETSU, Johnson City, TN 37614, USA

**Keywords:** Organic chemistry, Pharmaceutical chemistry

## Abstract

Cancer remains the second major cause of death in the world. Thus, there is a pressing need to identify potential synthetic route for the development of novel anticancer agents which will serve as lead compounds to effectively combat this life-threatening epidemic. Pyrrolo[2,1-c][1,4]benzodiazepines (PBDs) have sparked a great interest as lead compounds because of their cancerostatic and anti-infective properties. The twisted molecular structure of PBD analogs provides both helical and chiral elements. In an effort to expand novel PBDs that interact with the key exocyclic amino group of the DNA-guanine base, we hypothesized that construction of a fused cyclic active system, would likely serve as an electrophilic site when compared to traditional electrophilic C11-N10 imine group. To examine our theory, we report herein the synthesis and cell viability/cytotoxicity of a series of PBD analogs using NCI-60 cell lines screening. Thus, compounds **1**–**13** were synthesized and fully characterized. The selected PBDs were found to have marginal inhibition of growth, up to 30%, for certain cell lines.

## 1 Introduction

Naturally occurring pyrrolobenzodiazepines (PBDs) were first discovered in the cell cultures of the Streptomyces species in the 1960s [1]. The first PBD molecule to be separated and characterized was anthramycin [2]. Since then, a number of naturally occurring PBDs have been isolated from the *Streptomyces sp.* which include sibiromycin and tomaymycin, among others [3, 4]. It is presumed that the PBD molecules were evolved by the Streptomyces species as a form of chemical defense; however, scientists have made use of their antibiotic properties for the treatment of cancer.

**Fig. 1:**
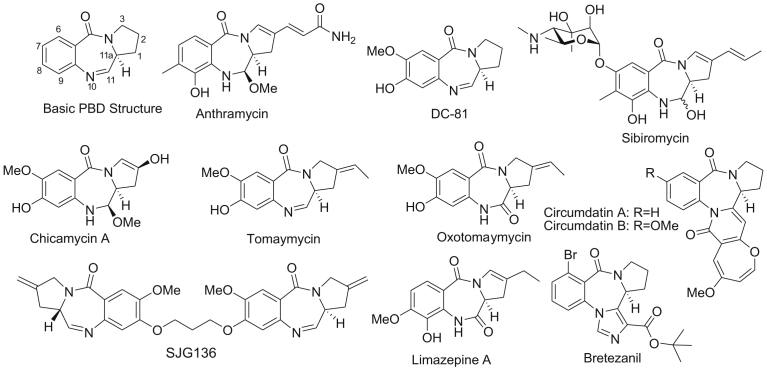
Naturally occurring PBDs and their synthetic analogs.

To date, thirteen natural PBD structures have been isolated from the Streptomyces species (Fig. 1) [5]. Of the thirteen PBD products that have been isolated, both anthramycin and sibiromycin were shown to have broad spectrum anti-tumor activity in-vitro and as a result, they have been tested clinically. Anthramycin particularly was found to have significant cytotoxicity against gastrointestinal, breast cancers, lymphomas and sarcomas without having significantly side effects towards red blood cells; however, due to its cardiotoxicity, anthramycin's clinical use was limited [6, 7].

Pyrrolobenzodiazepines are defined by their tricyclic structure, which is composed of an aromatic A-ring, a 1,4-diazepin-5-one B-ring, and a pyrrolidine C-ring. The PBD compounds differ from one another by the location and type of substituent groups on all three rings. The C-11a carbon has an S-configuration, which gives the molecules a right-handed twist. In addition to their right handed-twist, it is important to note that the natural PBDs can exist in three interchangeable forms at their N10-C11 position: imine, carbinolamine, and carbinolamine methyl ether [8]. The choice of solvent most certainly has an effect on which form exists in solution. However, the three interconvertible forms are normally considered to be equivalent. In all cases, the C11 position of the PBDs is electrophilic [9, 10].

PBDs have an *S*-configuration at carbon C11a which gives the molecules a right-handed twist. This right-handed twist allows the PBDs to fit snugly within the DNA double helix, enabling them to interact with the minor groove. In fact, a synthetic PBD with *R*-configuration at C11a was shown to lack DNA binding affinity and *in vitro* cytotoxicity. In combination with the right-handed twist of the molecules, electrophilic carbon atom at the C11 position allows the PBDs to alkylate the nucleophilic NH_2_ group of the guanine in the minor groove of DNA. Several studies have shown that the reaction between PBDs and DNA is sequence selective, preferentially targeting 5′-purine-G-purine sequences [11, 12]. The covalent bonds that the PBDs form to the amino group of the guanine base allows the molecules to act as an adduct that blocks biological processes such as transcription and RNA polymerase progression. The cytotoxic/antitumor activities of PBDs are attributed to these reactions.

Due to the efforts to synthesize PBDs which shared the characteristics of the relatively successful natural PBD products, a dimer by the name of SJG-136 was synthesized and was shown to be significantly cytotoxic *in vitro*. As a result of impressive *in vitro* studies, SJG136 was tested thoroughly *in vivo* against 10 human tumor xenografts, including but not limited to melanomas, ovarian carcinomas, breast cancer, colon cancer, and lung cancer. Due to the impressive and extensive data collected regarding SJG136's activity in pre-clinical trials, the compound entered clinical Phase I testing against advanced solid tumors in 2004. SJG-136, is presently in phase II clinical trials for treating patients with epithelial ovarian, primary peritoneal, or fallopian tube cancer. Furthermore, in the past decade there has been a growing interest in PBD dilactams where their N10-C11 amidic moiety have shown a tolerable robustness towards a number of synthetic transformations of the amide functionality to the DNA-alkylating imine as well as 5- and 6-membered fused heterocycles. Additionally, PBD dilactams and their tetracyclic analogs have presented interesting biological activity towards several different targets.

In continuation to our previous attempts to discover novel bioactive PBDs and improvement of their efficacy [13, 14, 15, 16], we became interested in the synthesis and characterization of a new class of tetracyclic PBD derivatives with fused heterocyclic moiety. We evaluated a panel of thirteen novel PBD's for *in vitro* cytotoxicity using NCI-60 cell lines screening.

## 2 Results and discussion

### 2.1 Chemistry

The synthesis of all PBD derivatives were accomplished with the readily available basic structure of PBD natural product (**1**) (from *Isatis indigotica* [17]) used as the starting material.

**Fig. 2:**
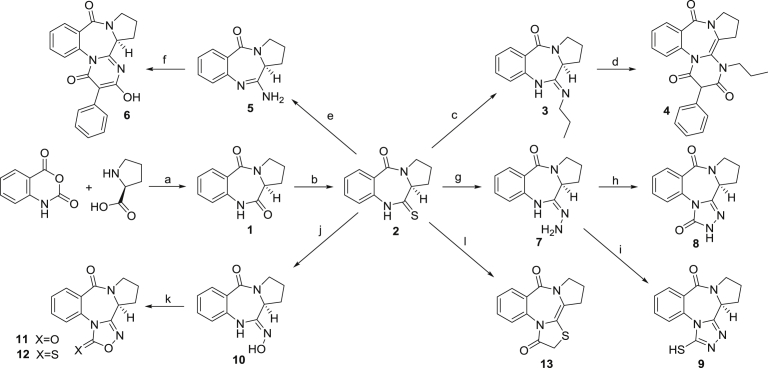
Reagents and conditions: (a) DMF, 155 °C, 5h, 82.0%; (b) Lawesson's reagent, THF, rt, 15h, 87.0%; (c) n-propylamine, HgCl_2_, rx, 1h, 88%; (d) bis(2,4,6-trichlorophenyl)-2-phenylmalonate, 190 °C, 10 min, 75%; (e) NH_3_(g)(anhy.), HgCl_2_, THF, rx, 1h, 86% (f) bis(2,4,6-trichlorophenyl)-2-phenylmalonate, 170–180 °C, 10 min, 63%; (g) N_2_H_4_.H_2_O (98%), EtOH(abs.), rt, 15h, 99.0%; (h) CDI, dioxane (anhy.), reflux, 24h, 99.0%; (i) TDI, dioxane (anhy.), rx, 24h, 80.0%; (j) NH_2_OH.HCl, K_2_CO_3_, ethanol, rt, 24h, 82%; (k) CDI, dioxane, rx, 12h, 88%/TDI, dioxane, rx, 12h, 90%; (l) α-bromoacetyl chloride, THF, rt, under N_2_(g), 15h, 75%. CDI: 1,1′-carbonyldiimidazole, TDI: 1,1′-thiocarbonyldiimidazole.

As depicted in the synthesis plan (Fig. 2), the cyclocondensation of equimolar mixture of L-proline and isatoic anhydride in DMF at 155 °C [18, 19] by a standard protocol afforded dilactam **1** which was recrystallized from a 10:1 v/v mixture of acetone and DMF to obtain compound **1** as off white crystals. Thionation of compound **1** with 0.5 equiv Lawesson's reagent in THF at room temperature afforded thiolactam **2** [13, 20].

Catalytic amination of thiolactam **2** using *n*-propylamine in the presence of mercuric(II)chloride as catalyst afforded compound **3** after being recrystallized from nitromethane. In the solid state, compound **3** exists as the exocyclic imine in the Z configuration, as evidenced by a single crystal X-ray analysis of a *N*-phenylimino analog [21]. The bond length of the C=N bond was determined to be shorter the N(10)–C(11) distance. The cyclization of compound **3** with bis (2,4,6-trichlorophenyl)-2-phenylmalonate at 190 °C using a *zincke* apparatus was performed under neat conditions to yield the pyrimidine-annulated PBD **4**. Cyclization reactions of *N*,*N*′-disubstituted amidines with bis-(2,4,6-trichlorophenyl)-2-phenylmalonates have generally been shown to result in the formation of pyridinium-4-olates. The loss of two molecules of trichlorophenol through a ketene intermediate during ring closure is thought to be one of the possible mechanistic explanations for the syntheses of these compounds [20, 21].

A standard protocol was followed for the treatment of **2** with hydrazine monohydrate in ethanol at room temperature to afford compound **7** in a quantitative yield which was further used as the main precursor for the syntheses of triazole-3-one **8** and mercapto-triazole **9** PBDs [22]. Carbonylation of **7** with 1,1′-carbonyldiimidazole (CDI) in dioxane under reflux resulted in the formation of **8**. Upon further purification by flash chromatography on silica gel column, pure **8** was formed as white crystals. Likewise, treatment of **7** with thiocarbonyldiimidazole (TDI) in dioxane under reflux produced **9**. Purification of crude product was performed on a silica gel column chromatography to yield pure **9** as yellow solid. Recrystallization from methanol afforded yellow single crystals in 80% overall yield. In contrast to the carbonylation of **7**, inspection of spectral data clearly revealed that thionylation of the substrate with TDI generated an aromatic triazole ring system with a thiol substitution (**9**) instead of a thionyl compound [22]. This observation is dedicated to the low electronegativity and larger atomic size of sulphur which consequently leads to the construction of an aromatic tautomer.

The synthetic procedure for compound **10** was optimized with some modification to the protocol previously reported by Rekowski *et al.* and Bartsch *et al.* Treatment of compound **2** with hydroxylamine hydrochloride under basic conditions led to the formation of **10**, which in turn was purified by recrystallization from nitromethane to give pure **10** as yellow needle-like crystals [23, 24]. Our experience showed that the use of K_2_CO_3_ as a relatively milder base rather than reported trimethylamine, significantly increased the reaction yield up to 94%. A possible explanation for this observation could be the ability of the K_2_CO_3_ to effectively neutralize the HCl and the gradual accessibility of hydroxylamine to attack by the electrophilic thionyl carbon in **2**.

Compound **10** was further used as the main precursor for the synthesis of oxadiazole-3-one **11** and oxadiazole-3-thione **12** by respective carbonylation and thiocarbonylation reactions [23, 24]. A slight modification was made to the reported protocol to develop more efficient syntheses of **11** and **12.** Treatment of **10** with CDI and TDI under reflux in anhydrous dioxane for 12 hours afforded **11** and **12** in 88% and 90% yield, respectively. According to the literature, both reactions in THF under reflux for 24 hours produce their respective products in 84% yields.

## 3 Biological activity

### 3.1 *In vitro* cytotoxicity

To determine whether our PBD compounds were cytotoxic towards cancer cells, nine PBD analogs were selected for the single-dose *in vitro* cytotoxicity using a panel of NCI-60 cell lines as part of Developmental Therapeutics Program (DTP) at the National Cancer Institute (Table 1). Compounds that selected for the NCI-60 cell screening were tested at a single concentration of 10 μM. All the compounds showed some non-selective growth inhibition on T-47D breast cell line ranging from 6.7 to 12.7%. Interestingly, PBDs **3** and **4** were displayed their highest inhibition on SNB-75 (CNS) (22.3%) and NCI-H522 (NSCL) (22.2%), respectively. Growth of the NCI-H522 cell line was inhibited by all the PBDs ranging from 9.5% to 23.7%. All nine PBDs inhibited growth on the renal cell line, UO-31, ranging from 10.8% to 28.5%. Overall the best inhibition activity was exhibited by PBD **6** which acts on the renal UO-31 cell line with 28.5% growth inhibition.

**Table 1:** *In vitro* cytotoxicity data of PBD derivatives against various NCI-60 cell lines.

Cancer	Cell line	PBD Compounds-Growth Percent								
		3	4	5	6	8	10	11	12	13
Leukemia	HL-60(TB)	101.38	103.22	103.05	101.26	101.25	90.92	103	94.58	87.83
	SR	102.79	103.91	95.32	103.31	92.26	99.34	103.8	91.96	101.39
NSCL	HOP-62	101.62	89.44	84.60	90.25	77.32	91.39	80.34	92.91	87.54
	NCI-H522	84.29	77.82	83.64	78.63	76.35	79.92	90.49	76.82	81.50
Colon	HCT-116	100.33	96.01	96.12	99.44	98.34	98.34	101.09	95.49	88.64
	HT29	97.57	99.46	101.60	95.26	98.65	94.64	95.04	99.63	91.31
CNS	SNB-75	77.68	87.15	83.05	84.45	88.78	93.52	89.29	90.43	85.08
	U251	98.25	99.23	91.64	98.40	95.70	96.42	96.22	95.79	96.22
Melanoma	SKMEL-2	95.73	91.26	96.42	96.80	90.48	91.71	97.71	82.08	94.75
	MALME-3M	92.15	95.68	97.19	94.85	88.95	95.21	83.85	98.70	100.22
Ovarian	NCI/ADR-RES	97.24	95.76	102.94	95.62	104.82	100.80	99.31	104.39	100.73
	SK-OV-3	103.74	98.55	95.20	106.55	88.06	101.62	88.46	92.95	90.84
Renal	UO-31	85.88	83.90	78.66	71.55	89.20	86.24	88.38	87.04	80.50
	TK-10	92.55	94.15	94.62	90.33	96.45	92.33	95.19	96.05	98.39
Prostate	PC-3	98.28	95.79	89.08	90.37	95.76	95.37	97.71	90.70	88.45
Breast	T-47D	88.30	88.54	89.48	91.74	83.60	92.91	93.26	91.42	87.23
	HS 578T	94.34	98.16	92.43	94.98	96.69	99.98	98.31	96.79	94.58

### 3.2 MTT cell viability test

**Fig. 3:**
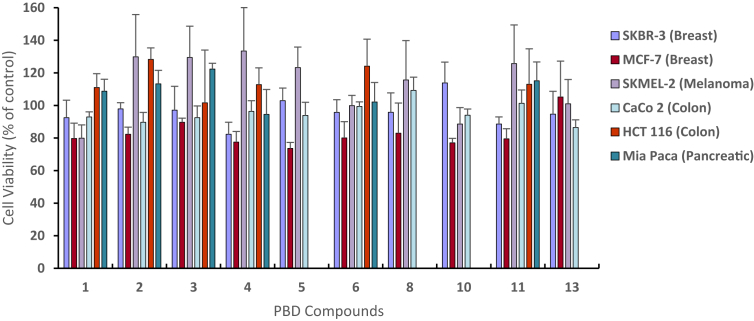
*In vitro* cytotoxicity assay of PBD analogs from MTT Assay.

To further examine our preliminary cytotoxicity results from NCI-60 cell lines in higher concentrations, the MTT in house assay was used for the selected cell lines. Similar results were obtained in agreement with the NCI-60 cell lines (Fig. 3). The PBD compounds were dosed at 100 μM, and allowed to incubate for 48 hours. Based on the previously reported works, the preferred cell lines that were used for the MTT assay were breast (SK-BR-3, MCF-7), colon (CaCo-2, HCT 116), melanoma (SKMEL-2), and pancreatic (Mia Paca) cell lines. Unlike other biological activity assays, we did not have access to the reference compound (positive control) for *in vitro* cytotoxicity/cell viability tests. Basically we cannot use the commercially available anti-cancer drugs because they possess a different target than BPD derived compounds. The only available potent antitumor agents are natural products such as anthramycin, tomaymycin, and sibiromycin which are either very expensive to purchase or too complicated to synthesize.

The results show that nearly all of the novel PBD compounds, except for compound **13**, reduced the cell viability of MCF-7 cells by roughly up to 27% (Fig. 3). In contrast, virtually none of the PBD analogs, with the exception of compounds **1** and **10**, had an anti-proliferative effect on the SKMEL-2 cell line. Additionally, all compounds that were tested on HCT-116 and Mia Paca showed no cytotoxic effects. The PBD **1** showed a decrease in cell viability for the SKBR-3, MCF-7, SKMEL-2, and CaCo 2 cell lines; however, the PBD dilactam showed no cytotoxicity towards HCT 116 or Mia Paca. The greatest amount of cytotoxicity for compound **1** was a reduction of cell viability by 20.3% and 20.1% for both MCF-7 and SKMEL-2 cell lines, respectively. PBD **2** showed a clear reduction of cell viability for MCF-7 and CaCo 2, while having the greatest amount of anti-proliferative effects on the MCF-7 cell line (17.7%). However, PBD **2** did not noticeably decrease the cell viability of cell lines SKBR-3, SKMEL-2, HCT 116, or Mia Paca. PBD **3** had a relatively low effect on the cell viabilities of MCF-7 (10.4%) and CaCo 2 (7.5%) cells, while had no negative effect on the cell growth of SKBR-3, Mia Paca, SKMEL 2, and HCT 116 cell lines. PBD **4** had a noticeable effect on the cell viabilities of SKBR-3 (17.3%) and MCF-7 (22.5%). However, PBD **4** had no negative effects on the cell growth of SKMEL-2, CaCo 2, HCT 116, or Mia Paca cell lines. PBD **5** showed the highest growth inhibition against MCF-7 cell line (26.4%) when compared with other tested analogs. PBD **5** had no negative affect on the cell viability of SKBR-3, SKMEL-2, and CaCo 2. PBDs **6** and **8** displayed some reduction in the cell viability of SKMEL-2 with values of 19.9% and 17.0%, respectively. PBDs **10** and **11** showed their highest inhibition activity versus SKMEL-2 breast cell line with 13.0% and 10.5%, respectively. PBD **13** had a selective anti-proliferative effect on CaCo 2 cell line (13.6%).

## 4 Conclusion

To synthesize PBDs **1**–**13** with side chain functionality at C11-N10 imino group, we either employed a new approach or developed a shorter and more efficient syntheses than those previously reported. The novel synthetic PBD's evaluated in this study demonstrated a range of biological activities similar to what has been described for the various naturally occurring PBDs. Among these activities, we saw a promising specificity when assaying the cytotoxic activity of nine of the synthesized PBDs where all showed some inhibition of growth on the renal cell line UO-31 with PBD **6** showing the greatest efficacy. PBD **8** also showed inhibition of growth in both the HOP-62 and NCI-H522 cell lines. Additional investigation of the cell viability/cytotoxicity of these compounds, as well as development of derivatives of the studied PBDs, is in progress.

## 5 Experimental

**General Experimental Procedures.** All reactions proceeded from the commercially available L-proline and isatoic anhydride. Unless otherwise stated, all reagents and solvents were obtained from commercial vendors and utilized without further purification. ^1^H NMR, ^13^C NMR, GC-MS and IR spectroscopy were the techniques used for the characterization of the synthesized compounds. JEOL-NMR Eclipse-400 MHz spectrophotometer provided all NMR data and spectra. The high frequency position conversion gave the different chemical shifts of all peaks in parts per million (ppm) with the coupling constants value (J) reported in Hz. The splitting patterns of resonance were also described as follows: singlet (s), doublet (d), doublet of doublet (dd), doublet of doublet of doublet (ddd), triplet (t), quartet (q), and multiplet (m). Genesis II FT-IR spectrometer was also used for all IR spectra. Cambridge Melt-Temp device provided melting point readings but without correction for the synthesized compounds. Shimadzu GC-MS 2010 System provided the relative abundances of the smaller ion fragments from the molecular ion of all compounds. Other common chromatographic techniques such as thin layer chromatography and column chromatography were also employed in the purification of all synthesized compounds. Compounds **5** and **6** were synthesized according to previously reported methods [15].

*(S)-1,2,3,11a-tetrahydro-5H-benzo[e]pyrrolo[1,2-a][1,4] diazepine-5,11(10H)-dione (****1****).* Dilactam (**1**) was synthesized according to the previously reported methods with some modifications [17]. A suspension of isotaic anhydride (10.0 g, 61.34 mmol) and L-proline (7.06 g, 61.34 mmol) in *N*,*N*-dimethylformamide (DMF) (50 mL) was heated to 155 °C for 5 h. After cooling to room temperature, the solvent was removed in vacuo and the residue was taken up in water. The precipitate was collected and dried to give an off-white solid. Recrystallization from Acetone/DMF (10:1 v/v) afforded 10.87 g (82%) of pure compound **1** as off-white crystals. Yield: 82.0%; m.p: 223–225 °C; [α]^25^_D_ = +512° (c 0.5, MeOH); ^1^H-NMR (400 MHz, DMSO-d6): δ = 1.80 (d, *J* = 9.9 Hz, 1H), 1.85–2.07 (m, 2H), 3.39–3.52 (m, 1H), 3.51–3.67 (m, 1H), 4.02–4.21 (m, 1H), 7.07–7.17 (m, 1H), 7.17–7.27 (m, 1H), 7.51 (ddd, *J* = 8.6, 6.8, 1.3 Hz, 1H), 7.78 (dd, *J* = 7.9, 1.6 Hz, 1H), 10.52 (s, 1H); ^13^C-NMR (100 MHz, DMSO-d6): δ = 23.6 (C-2), 26.3 (C-1), 47.4 (C-3), 56.8 (C-11a), 121.8, 124.4, 127.1, 130.8, 132.6, 136.9, 165.0 (CO), 171.3 (CO); IR (KBr) $\tilde{\nu }$ (cm^−1^) = 3222 (N-H), 3206, 2955, 2918, 2850, 1691 (C=O), 1680 (C=O), 1621, 1551, 1536, 1479, 1443, 1412, 1385, 1285, 1259, 1179, 759, 701, 615; GC-MS (70 eV) m/z (%): 216 (10) [M+], 119 (14), 92 (20), 70 (100), 64 (10); HRMS m/z calcd for C_12_H_13_N_2_O_2_ [M+H]^+^ 217.0977, found 217.0982.

*(S)-11-thioxo-1,2,3,10,11,11a-hexahydro-5H-benzo[e]pyrrolo [1,2-a][1,4]diazepin-5-one (****2****).* A mixture of compound **1** (21.60 g, 100 mmol) and Lawesson's reagent (20.2 g, 50 mmol) in THF (400 mL) was stirred over night at room temperature. Evaporation of solvent in vacuo gave a crude solid yellow residue. The solid was washed with toluene and further washed with cold toluene to afford 20.2 g (87%) of pure compound **2** as yellow solid [17]. Yield: 87.0%; m.p: 272–274 °C; [α]^25^_D_ = +762° (c 0.5, CHCl_3_); ^1^H-NMR (400 MHz, DMSO-d6): δ = 1.83–1.98 (m, 1H), 1.98–2.15 (m, 2H), 2.88 (d, *J* = 5.9 Hz, 1H), 3.31–3.51 (m, 3H), 3.53–3.60 (m, 1H), 4.28 (d, *J* = 6.2 Hz, 1H), 7.29 (d, J = 8.1 Hz, 1H), 7.32–7.39 (m, 1H), 7.58 (td, J = 7.7, 1.2 Hz, 1H), 7.83 (dd, *J* = 7.7, 1.5 Hz, 1H); ^13^C-NMR (100 MHz, DMSO-d6): δ = 23.2 (C-2), 29.5 (C-1), 47.4 (C-3), 60.3 (C-11a), 122.3, 126.2, 128.3, 130.8, 132.7, 137.0, 164.7 (CO), 202.5 (CS); IR (KBr) $\tilde{\nu }$ (cm^−1^) = 3125 (N-H), 3094, 3063, 3024, 2974, 1620 (C=O), 1579, 1523, 1478, 1452, 1418, 1381, 1272, 1193, 1166, 1145, 1103, 1069, 1055, 887, 833, 817, 786, 755, 695, 664, 625; GC-MS (70 eV) m/z (%): 232 (7) [M+], 108 (6), 70 (100), 68 (6); HRMS m/z calcd for C_12_H_13_N_2_OS [M+H]^+^ 233.0749, found 233.0737.

*(S,Z)-11-(propylimino)-1,2,3,10,11,11a-hexahydro-5H-benzo[e]pyrrolo[1,2-a][1,4]diazepin-5-one (****3****).* To a stirred suspension of compound **2** (5.80 g, 25.0 mmol) and propylamine (20 mL) was added HgCl_2_ (7.14 g, 26.25 mmol) at 60 °C. The mixture was stirred for further 1 h at this temperature. After cooling to room temperature, the mixture was filtered through a plug of celite and eluted with dichloromethane (CH_2_Cl_2_) [13]. The filtrate was washed with saturated Na_2_S_2_O_3_ and after extraction with CH_2_Cl_2_, the combined organic layers were dried over magnesium sulfate (MgSO_4_), filtered, and the solvent and excess amine were evaporated under reduced pressure. The crude solid was purified by recrystallization in nitromethane to afford 5.64 g of pure compound **3** as colorless crystals. Yield: 88.0%; m.p: 159–161 °C; [α]^25^_D_ = +1106° (c 0.5, CHCl_3_); ^1^H-NMR (400 MHz, CDCl_3_): δ = 0.98 (t, *J* = 7.5 Hz, 3H), 1.48–1.72 (m, 2H), 2.03 (q, *J* = 8.9 Hz, 1H), 2.12 (d, *J* = 7.0 Hz, 1H), 2.18–2.29 (m, 2H), 3.38 (d, *J* = 4.8 Hz, 2H), 3.50–3.64 (m, 1H), 3.82–3.93 (m, 1H), 4.03 (t, *J* = 4.9 Hz, 1H), 4.69 (s, 1H), 7.02–7.13 (m, 2H), 7.38 (ddd, *J* = 8.6, 6.8, 1.3 Hz, 1H), 7.92 (dd, *J* = 7.7, 1.5 Hz, 1H); ^13^C-NMR (100 MHz, CDCl_3_): δ = 11.7 (CH_3_), 22.3 (CH_2_), 23.9 (CH_2_), 26.8 (C-2), 43.3 (C-1), 46.5 (C-3), 54.4 (C-11a), 122.2, 126.5, 126.9, 130.1, 131.6, 146.7, 156.2 (C-N), 166.8 (CO); IR (KBr) $\tilde{\nu }$ (cm^−1^) = 3851, 3798, 3745, 3356 (N-H), 3319 (N-H), 3287, 3061, 2941, 2880, 2815, 2359, 2328, 1826, 1791, 1731, 1605 (C=O), 1554, 1531, 1506, 1456, 1406, 1383, 1336, 1256, 1215, 1150, 1096, 1067, 1035, 991, 918, 835, 761, 703, 635; GC-MS (70 eV) m/z (%): 257 (21) [M+], 146 (23), 119 (23), 90 (21), 70 (100); HRMS m/z calcd for C_15_H_20_N_3_O [M+H]^+^ 258.1606, found 258.1629.

*3-Phenyl-1-propyl-1,12,13,14,14a,14b-hexahydro-2H,10H-benzo[e]pyrimido[2,1-c]pyrrolo[1,2-a][1,4]diazepine-2,4,10(3H)-trione (****4****).* A mixture of **3** (1 mmol, 0.257 g) and bis(2,4,6-trichloro phenyl)-2-phenylmalonate (1 mmol, 0.539 g) was heated at 190 °C for 10 minutes in a Zincke apparatus under high vacuum. The residue was treated with diethyl ether to give a dark brown precipitate which was collected by filtration and washed with diethyl ether. Recrystallization was done in 2-propanol. Yield: 300 mg (75%) m.p.: 230–233 °C; [α]^25^_D_ = 0° (c = 0.5, CHCl_3_); ^1^H-NMR (400 MHz, CDCl_3_): δ = 0.77–0.81 (t, *J* = 7.3 Hz, 3H), 1.31–1.39 (m, 2H), 2.04–2.17 (m, 1H), 2.21–2.28 (m, 1H), 2.62–2.71 (m, 1H), 2.77–2.82 (dd, *J* = 14.6, 6.2 Hz, 1H), 2.96–3.03 (m, 1H), 3.96–4.11 (m, 2H), 4.17–4.25 (m, 1H), 4.71 (s, 1H), 7.28–7.30 (m, 2H), 7.36–7.45 (m, 5H), 7.54–7.59 (td, *J* = 7.8, 1.6 Hz, 1H), 8.02–8.04 (m, 1H); ^13^C-NMR (100 MHz, CDCl_3_): δ = 11.2, 20.6, 21.5, 29.6, 48.4, 49.5, 59.1, 118.0, 125.5, 127.5, 128.2, 128.4, 130.8, 131.3, 132.3, 133.3 (CO), 139.8 (CO), 167 (CO); IR (KBr): $\tilde{\nu }$ (cm^−1^) = 2957, 2918, 2860, 2355, 1958, 1728, 1683 (C=O), 1633 (C=O), 1576, 1487, 1453, 1352, 1297, 1252, 1221, 1185, 1150, 899, 800, 753, 719, 698, 665, 631; UV λmax (AcO): 194, 274 nm; GC-MS (70 eV) m/z (%): 401 (66) [M+], 215 (11), 187 (29), 118 (100), 90 (46); HRMS m/z calcd for C_24_H_23_N_3_O_3_ [M+H]^+^ 402.1812, found 402.1801.

*(S,E)-11-amino-1,2,3,10,11,11a-hexahydro-5H-benzo[e]pyrrolo[1,2-a][1,4]diazepin-5-one (****5****).* To a stirred suspension of monothiolactam 2 (1.16 g, 5.0 mmol) in anhydrous THF (50 mL) was added HgCl_2_ (1.63 g, 6.0 mmol) and pure anhydrous ammonia was bubbled through the mixture for 1 h at 60 °C. After cooling, the resulting suspension was filtered off through a pad of Celite and eluted with methanol. The solvent was evaporated under reduced pressure. The resultant solid was purified by recrystallization from anisole/methanol to afford pure colorless crystals [14]. Yield: 0.925 g (86%); m.p.: >235 °C (dec.); [α]^25^_D_ = +515° (*c* = 0.5 in MeOH); ^1^H-NMR (400 MHz, DMSO-d_6_): δ = 1.93–2.04 (m, 2H), 2.10–2.19 (m, 1H), 2.43–2.59 (m, 1H), 3.35–3.42 (m, 1H), 3.63–3.69 (m, 1H), 4.25 (d, *J* = 7.8 Hz, 1H), 7.15 (d, *J* = 7.5 Hz, 1H), 7.24 (t, *J* = 7.5 Hz, 1H), 7.30–7.72 (br s, 2H, NH_2_), 7.51–7.56 (m, 1H), 7.81 (dd, *J* = 7.8, 1.5 Hz, 1H); ^13^C-NMR (100 MHz, DMSO-d_6_): δ = 24.0 (C-2), 26.7 (C-1), 47.4 (C-3), 55.1 (C-11a), 124.6, 125.1, 127.7, 131.0, 132.8, 141.3, 164.3 (C-N), 165.4 (CO); IR (KBr): $\tilde{\nu }$ (cm^−1^) = 3356 (N-H), 3125 (N−H), 1614 (C=O), 1578, 1456, 1239; GC-MS (70 eV) m/z (%): 215 (39) [M^+^], 70 (100); HRMS m/z calcd for C_12_H_14_N_3_O [M+H]^+^ 216.1137, found 216.1139.

*(S)-3-phenyl-1,12,13,14,14a,14b-hexahydro-2H,10H-benzo[e]pyrimido[2,1-c]pyrrolo[1,2-a][1,4]diazepine-2,4,10(3H)-trione (****6****).* A mixture of **5** (1 mmol, 0.215 g) and bis(2,4,6-trichlorophenyl) 2-phenylmalonate (1 mmol, 0.539 g) was heated at 170–180 °C for 10 minutes in a Zincke apparatus under high vacuum. The residue was treated with diethyl ether to give a dark brown precipitate which was collected by filtration and washed with diethyl ether. Recrystallization was performed in DMF/Water. Yield: 228 mg (63 %); m.p.: >250 °C (dec.); [α]^25^_D_ = −34° (c = 0.5 in MeOH); ^1^H-NMR (400 MHz, DMSO-d_6_): δ = 1.92–1.97 (m, 1H), 2.01–2.14 (m, 2H), 2.75–2.77 (m, 1H), 3.37–3.44 (m, 1H), 3.58–3.69 (m, 1H), 4.50 (d, *J* = 6.3 Hz, 1H), 7.12–7.15 (m, 1H), 7.21–7.27 (m, 4H), 7.48 (d, *J* = 8.0 Hz, 1H), 7.52–7.55 (m, 1H), 7.59–7.64 (m, 1H), 7.80 (dd, *J* = 7. 5, 1.1 Hz, 1H), 11.71 (s, 1H). ^13^C-NMR (100 MHz, CDCl_3_): δ = 24.1 (C-2), 27.0 (C-1), 47.0 (C-3), 58.9 (C-11a), 101.6, 126.6, 129.0, 129.2, 129.3, 129.7, 129.8, 131.1, 132.6, 133.8, 141.4, 158.0, 163.8, 164.2, 164.5 (C=O); IR (KBr): $\tilde{\nu }$ (cm^−1^) = 1667, 1626, 1604, 1558, 1458; GC-MS (70 eV) m/z (%): 359 (100) [M^+^]; HRMS m/z calcd for C_21_H_18_N_3_O_3_ [M+H]^+^ 360.1348, found 360.1365.

*(S,E)-11-hydrazono-1,2,3,10,11,11a-hexahydro-5H-benzo[e]pyrrolo[1,2-a][1,4]diazepin-5-one (****7****).* To a solution of compound **2** (1.16 g, 5.0 mmol) in ethanol (20 mL) was added 98% hydrazine monohydrate (0.75 g, 15.0 mmol) and stirred for 15 h at room temperature. The solvent was removed in vacuo and the residue was taken up in water. The precipitate was collected, dried and washed with diethyl ether to afford 1.14 g (99%) of compound **7** as off-white solid [15]. Yield: 99.0%; m.p: 178–180 °C; [α]^25^_D_ = +552° (c 0.5, MeOH); ^1^H-NMR (400 MHz, CDCl_3_): δ = 1.61–2.08 (m, 4H), 2.65–2.82 (m, 1H), 3.57–3.69 (m, 1H), 3.71–3.84 (m, 1H), 4.17–4.30 (m, 1H), 6.85 (d, *J* = 8.1 Hz, 1H), 7.09 (t, *J* = 7.7 Hz, 1H), 7.32–7.40 (m, 1H), 7.90 (dd, *J* = 7.9, 1.3 Hz, 1H); ^13^C-NMR (100 MHz, CDCl_3_): δ = 23.4 (C-2), 26.1 (C-1), 47.3 (C-3), 55.5 (C-11a), 119.7, 123.0, 125.4, 131.5, 132.5, 137.8, 152.2, 166.2 (CO); IR (KBr): 3484, 3282, 3240, 3161, 2967, 2949, 2871, 2814, 2422, 2358, 2336, 1957, 1854, 1784, 1766, 1726, 1689, 1658, 1619, 1563, 1539, 1529, 1479, 1442, 1384, 1323, 1272, 1202, 1076, 943, 903, 825; HRMS m/z calcd for C_12_H_15_N_4_O [M+H]^+^ 231.1246, found 231.1239.

*(S)-11,12,13,13a-tetrahydro-9H-benzo[e]pyrrolo[1,2-a][1,2,4]triazolo[3,4-c][1,4]diazepine-3,9(2H)-dione (****8****).* A solution of compound **7** (0.69 g, 3.0 mmol) and 1,1′-carbonyldiimidazole (3.89 g, 24.0 mmol) in dioxane (25 mL) was refluxed for 24 h. The solvent was removed in vacuo and the crude product was purified by flash gel column chromatography using gradient elution. The ratios of solvent mixture used were EtOAc/Hexane (1:1, 2:1, 4:1 v/v) and a final elution with only Ethyl acetate (EtOAc). Recrystallization from EtOAc/Hexane (4:1 v/v) afforded 0.76 g (99%) of pure compound **8** as white crystals. Yield: 99.0%; m.p: 238–240 °C; [α]^25^_D_ = +136° (c = 0.5, DMSO); ^1^H-NMR (400 MHz, CDCl_3_): δ = 1.99–2.22 (m, 2H), 2.23–2.37 (m, 1H), 2.86 (ddd, *J* = 16.3, 6.8, 3.7 Hz, 1H), 3.64–3.75 (m, 1H), 3.81–3.92 (m, 1H), 4.53 (dd, *J* = 8.4, 3.3 Hz, 1H), 7.42–7.51 (m, 1H), 7.59–7.67 (m, 1H), 7.90–7.98 (m, 1H), 8.03 (dd, *J* = 7.9, 1.6 Hz, 1H), 9.90 (s, 1H); ^13^C-NMR (100 MHz, CDCl_3_): δ = 23.5 (C-2), 26.0 (C-1), 47.7 (C-3), 51.7 (C-11a), 123.0, 127.8, 129.0, 130.1, 131.9, 132.3, 153.9 (CO), 164.7 (CO); IR (KBr): 3435, 3375, 3183, 3091, 3000, 2909, 2878, 2814, 2326, 1711, 1611, 1574, 1490, 1466, 1412, 1343, 1285, 1239, 1202, 1167, 1118, 1072, 1024, 979, 926, 912, 825, 786, 758, 709, 656, 616; GC-MS (70 eV) m/z (%): 256 (60) [M+], 102 (26), 90 (38), 43 (100); HRMS m/z calcd for C_13_H_12_N_4_O_2_ [M+H]^+^ 257.1033, found 257.1012.

*(S)-3-mercapto-11,12,13,13a-tetrahydro-9H-benzo[e]pyrrolo[1,2-a][1,2,4]triazolo[3,4-c][1,4]diazepin-9-one (****9****).* A solution of compound **7** (0.23 g, 1.0 mmol) and 1,1′-thiocarbonyldiimidazole (1.42 g, 8.0 mmol) in dioxane (20 mL) was refluxed for 24 h. The solvent was removed in vacuo and the crude product was purified by flash gel column chromatography using gradient elution. The ratios of solvent mixture used were Acetone/Hexane (10:1, 20:1 v/v). Recrystallization from EtOAc/Hexane (4:1 v/v) afforded 0.217 g (80%) of pure compound **9** as brown crystals. Yield: 80.0%; m.p: >230 °C (dec.); [α]^25^_D_ = +37.5° (c 0.4, CHCl_3_); ^1^H-NMR (400 MHz, CDCl_3_): δ = 1.62–1.99 (m, 1H), 2.03–2.28 (m, 2H), 2.29–2.45 (m, 1H), 2.89 (tt, *J* = 9.9, 3.4 Hz, 1H), 3.63–3.78 (m, 1H), 3.81–3.97 (m, 1H), 4.55 (dd, *J* = 8.4, 2.9 Hz, 1H), 7.50–7.60 (m, 1H), 7.63–7.77 (m, 1H), 8.04 (dd, *J* = 7.7, 1.5 Hz, 1H), 8.39 (d, *J* = 8.1 Hz, 1H); ^13^C-NMR (100 MHz, DMSO-d6): δ = 23.6 (C-2), 26.7 (C-1), 47.5 (C-3), 51.6 (C-11a), 126.2, 129.1, 130.6, 130.8, 131.2, 131.4, 153.1, 164.0 (CO), 167.7 (CS); IR (KBr) $\tilde{\nu }$ (cm^−1^) = 3735, 3280, 3174, 3127, 3096, 3060, 2989, 2944, 2880, 2827, 2415, 2359, 2331, 1958, 1760, 1723, 1686, 1611, 1542, 1488, 1410, 1344, 1310, 1286, 1216, 1112, 1043, 1003, 973, 921, 892, 876, 825, 756, 704, 664, 638, 622, 610; GC-MS (70 eV) m/z (%): 272 (100) [M+], 116 (23), 90 (34), 70 (33); HRMS m/z calcd for C_13_H_12_N_4_O_1_S [M+H]^+^ 273.0805, found 273.0833.

*(S,E)-11-(hydroxyimino)-1,2,3,10,11,11a-hexahydro-5H-benzo[e]pyrrolo[1,2-a][1,4]diazepin-5-one (****10****).* To a solution of compound **2** (1.16 g, 5.0 mmol) in ethanol (30 mL) was added the hydroxylamine hydrochloride (0.417 g, 6.0 mmol) and potassium carbonate (2.20 g, 16.0 mmol). The light yellow mixture was stirred for 24 h at room temperature, where upon the mixture decolorized and H_2_S was released. After extraction with dichloromethane (100 mL) and washed with water (60 mL), the organic layer was further washed with brine, dried over sodium sulfate (Na_2_SO_4_), filtered and the solvent was removed in vacuo. The crude residue was recrystallized from nitromethane to afford 0.95 g (82%) of pure compound **10** as off-white crystals [21]. Yield: 82.0%; m.p: 150–153 °C; [α]^25^_D_ = +488° (c 0.5, CHCl_3_); ^1^H-NMR (400 MHz, CDCl_3_): δ = 1.68 (d, *J* = 11.7 Hz, 1H), 1.89–2.19 (m, 4H), 2.59–2.73 (m, 1H), 3.57–3.74 (m, 1H), 3.76–3.86 (m, 1H), 4.33 (dd, *J* = 7.5, 2.0 Hz, 1H), 6.90 (d, *J* = 7.3 Hz, 1H), 7.09–7.18 (m, 1H), 7.39–7.46 (m, 1H), 7.76 (s, 1H), 7.93 (dd, *J* = 7.9, 1.6 Hz, 1H); ^13^C-NMR (100 MHz, CDCl_3_): δ = 23.2 (C-2), 25.9 (C-1), 47.4 (C-3), 54.4 (C-11a), 120.5, 123.5, 125.8, 131.6, 132.6, 136.9, 151.2, 166.2 (CO); IR (KBr): 3788, 3716, 3281, 2965, 2911, 2878, 2806, 2351, 1724, 1689, 1658, 1612, 1573, 1552, 1530, 1480, 1453, 1425, 1396, 1273, 1227, 1201, 1162, 1108, 1040, 997, 957, 933, 884, 847, 807, 787, 756, 701, 663; GC-MS (70 eV) m/z (%): 231 (20) [M+], 144 (37), 90 (35), 70 (100); HRMS m/z calcd for C_12_H_14_N_3_O_2_ [M+H]^+^ 232.1086, found 232.1095.

*(S)-11,12,13,13a-tetrahydro-3H,9H-benzo[e][1,2,4]oxadiazolo [3,4-c]pyrrolo[1,2-a][1,4]diazepine-3,9-dione (****11****).* To a solution of compound **10** (231 mg, 1 mmol) in anhydrous dioxane (8 mL) was added 1,1′-carbonyldiimidazole (486.45 mg, 3.3 mmol) under nitrogen. The reaction mixture was refluxed for 12 h and the solvent removed in vacuo afterward. The residue was taken up in dichloromethane and washed three times with water. The organic layer was dried over Na_2_SO_4_ and the solvent removed in vacuo. The crude residue was purified by flash column chromatography to obtain a white solid which was recrystallized to afford compound **11** as crystals [21]. Yield: 226.16 mg (88 %); m.p.: 180–182 °C; [α]^25^_D_ = +142° (c = 0.5, CDCl_3_); ^1^H-NMR (400 MHz, CDCl_3_): δ = 1.58 (d, *J* = 12.1 Hz, 1H), 2.10–2.23 (m, 2H), 2.30–2.39 (m, 1H), 3.67–3.74 (m, 1H), 3.90–3.95 (m, 1H), 4.58–4.61 (dd, *J* = 8.4, 2.9 Hz, 1H), 7.49–7.53 (m, 1H), 7.64–7.68 (td, *J* = 7.8, 1.6 Hz, 1H), 7.83–7.85 (d, *J* = 7.3 Hz, 1H), 8.02–8.04 (dd, *J* = 7.9, 1.6 Hz, 1H); ^13^C-NMR (100 MHz, CDCl_3_): δ = 23.4, 25.6, 47.9, 51.2, 122.4, 128.5, 128.6, 128.7, 132.8, 156.3 (CN), 158.0 (CO), 164.2 (CO); IR (KBr) $\tilde{\nu }$ (cm^−1^) = 3623, 3335, 3044, 2956, 2918, 2875, 2851, 2381, 2349, 2296, 2199, 2105, 1981, 1838, 1787, 1728, 1710, 1690, 1657, 1640, 1599, 1551, 1468, 1451, 1410, 1301, 1267, 1167, 1081, 1025, 990, 761, 702, 662, 608; UV λmax (MeOH): 198, 274 nm; GC-MS (70 eV) m/z (%): 257 (22) [M+], 144 (100), 116 (38), 90 (33), 44 (33), 41 (24); HRMS m/z calcd for C_13_H_12_N_3_O_3_ [M+H]^+^ 258.0879, found 258.0871.

*(S)-3-thioxo-11,12,13,13a-tetrahydro-3H,9H-benzo[e][1,2,4] oxadiazolo[3,4-c]pyrrolo[1,2-a][1,4]diazepin-9-one (****12****).* To a solution of compound **10** (231 mg, 1 mmol) in anhydrous dioxane (8 mL) was added 1,1′-thiocarbonyldiimidazole (486 mg, 3.3 mmol) under nitrogen. The reaction mixture was refluxed for 12 h and the solvent removed in vacuo afterward. The residue was taken up in dichloromethane and washed three times with water. The organic layer was dried over Na_2_SO_4_ and the solvent removed in vacuo. The crude residue was purified by flash column chromatography to obtain a pale yellow solid and further recrystallized using hexane: ethyl acetate (1:1 v/v) to afford compound **12** as yellow crystals. Yield: 245.7 mg (90 %); m.p.: 216–218 °C; [α]^25^_D_ = +34° (c = 0.5, CDCl_3_); ^1^H-NMR (400 MHz, CDCl_3_): δ = 1.17–1.27 (m, 1H), 2.06–2.29 (m, 2H), 2.32–2.47 (m, 1H), 3.65–3.72 (m, 1H), 3.90–3.95 (m, 1H), 4.57–4.60 (dd, *J* = 8.6, 2.7 Hz, 1H), 7.55–7.59 (td, *J* = 7.6, 0.9 Hz, 1H), 7.67–7.72 (m, 1H), 8.01–8.04 (dd, *J* = 8.1, 1.5 Hz, 1H), 8.27–8.29 (dd, *J* = 8.2, 0.9 Hz, 1H); ^13^C-NMR (100 MHz, CDCl_3_): δ = 23.5, 26.5, 47.6, 51.0, 124.7, 129.6, 129.7, 131.8, 132.2, 159.1 (CN), 164.5 (CO), 164.8 (CS); IR (KBr) $\tilde{\nu }$ (cm^−1^) = 3788, 3716, 3281, 2965, 2911, 2878, 2806, 2351, 1724, 1689, 1658, 1612, 1573, 1552, 1530, 1480, 1453, 1425, 1396, 1273, 1227, 1201, 1162, 1108, 1040, 997, 957, 933, 884, 847, 807, 787, 756, 701, 663; UV λmax (MeOH): 198, 276 nm; GC-MS (70 eV) m/z (%): 273 (70) [M+], 146 (42), 102 (75), 90 (65), 69 (44), 43 (100); HRMS m/z calcd for C_13_H_12_N_3_O_2_S [M+H]^+^ 274.0650, found 274.0645.

*5,6-Dihydro-4H-3-thia-6a,11b-diazabenzo[g]cyclopenta [e]azulene-1,7-dione (****13****).* To a solution of compound **2** (0.464 g, 2.0 mmol) in anhydrous THF (40 mL) was added freshly distilled α-bromoacetyl chloride (0.39 g, 2.4 mmol). The mixture was stirred for 15 h at room temperature under nitrogen and then quenched by addition of saturated sodium bicarbonate (NaHCO_3_) (20 mL). After extraction with chloroform (2 × 20 mL), the combined organic layers were dried over Na_2_SO_4_, and the solvent was removed under reduced pressure. The crude residue was subjected to flash silica gel column chromatography using EtOAc/Hexane (4:1 v/v) as eluent and the solvent was evaporated under reduced pressure to give crude yellow solids [9]. The crude solid was purified by recrystallization from Ethanol/Water mixture to afford 0.20 g (75%) of pure compound **13** as yellow needle-like crystals. Yield: 75.0%; m.p: 165–167 °C; ^1^H-NMR (400 MHz, CDCl_3_): δ = 1.98–2.05 (m, 2H), 2.67 (t, *J* = 8.0 Hz, 2H), 3.83 (s, 2H), 3.90–3.94 (m, 2H), 7.28–7.32 (m, 1H), 7.44 (dd, *J* = 8.2, 1.0 Hz, 1H), 7.51–7.55 (m, 1H), 8.00 (dd, *J* = 8.0, 1.6 Hz, 1H); ^13^C-NMR (100 MHz, CDCl_3_): δ = 20.8 (C-5), 31.1 (C-4), 35.8 (C-2), 50.1 (C-6), 115.5, 124.3, 124.9, 127.2, 128.5, 133.3, 133.6, 138.9, 165.4 (CO), 172.7 (CO); IR (KBr) $\tilde{\nu }$ (cm^−1^) = 3398, 3357, 3282, 2944, 2880, 2790, 2419, 2385, 2350, 2323, 1856, 1818, 1779, 1724, 1690, 1582, 1482, 1404, 1384, 1358, 1334, 1217, 1126, 1092, 871, 835, 776, 708, 659, 636; HRMS m/z calcd for C_14_H_13_N_2_O_2_S [M+H]^+^ 273.0698, found 273.0706.

### 5.1 NCI 60 Human Tumor Cell Lines Screen

NCI-60 Human Tumor Cell Lines Screen is a part of the National Cancer Institute's (NCI) Development Therapeutics Program (DTP)-Drug Synthesis and Chemistry Branch. The screening utilizes 60 different human tumor cell lines to identify and characterize novel compounds with growth inhibition or cell death. The 60 human tumor cell lines represent leukemia, melanoma, and cancers of the lung, colon, brain, ovary, breast, prostate, and kidneys. The NCI-60 screening is free of cost to those who submit compounds; however, compounds submitted are reviewed and only those that meet the guidelines set by the NCI are selected for screening. Compounds that selected for the NCI-60 cell screening are initially dissolved in DMSO:glycerol 9:1 and tested at a single concentration of 10 μM. The One-dose data is reported in graphical form as a mean of the percent growth of treated cells (Fig. 3). The value reported for the One-dose assay of the individual compounds is cell growth relative to the no-drug control in addition to the initial number of tumor cells present, which allows for the detection of both growth inhibition (values between 0 and 100) and tumor cell death (values less than 0). Compounds that satisfy predetermined thresholds for the One-dose assay set by the NCI are tested by a Five-dose assay.

### 5.2 Cell culture maintenance

The cells lines that originated in multiple tissue types were obtained from the American Tissue Type Culture Collection (ATCC), including: breast (SK-BR-3, MCF-7), colon (Caco-2, HCT 116), melanoma (SKMEL-2), and pancreatic (Mia Paca) cell lines. The tumor cell lines were grown in their respective medium according to ATCC instructions. Each medium was supplemented with 10% serum and penicillin/streptomycin. Cell lines were allowed to reach 75% confluency before treatment with the novel pyrrolobenzodiazepines or negative control (DMSO at a final maximum concentration of 0.01%). The cell lines obtained from the ATCC were stored in liquid nitrogen until ready to use. Once the compounds were synthesized and ready to be tested, each cell line was allowed to thaw at room temperature, respectively. They were then transferred to a centrifuge tube, diluted by 5 mL of their respective media (listed on the ATTC website), and centrifuged for 3 minutes. The supernatant fluid was then removed, and the pellet was re-suspended in 10 mL of its media, and mixed thoroughly using a pipette. Finally, the solution was transferred to a 25-cm^2^ vented flask, and placed in an incubator. The incubator maintained an atmosphere of 37 °C and 5% carbon dioxide. The media inside the culture flask was exchanged with fresh media every few days in order to remove dead cells and provide fresh nutrients. The cells were allowed to grow until they reached 75% confluency. Once cells were 75% confluent, the media was removed from the flask and 1.5 mL of trypsin was added in order to lift the cells from the flask. The solution of cells were then transferred to a centrifuge tube, and diluted with 5 mL of media. Again, the solution was centrifuged for 3 minutes until all of the cells formed a pellet in the bottom of the tube. The supernatant fluid was then removed and replaced with 10 mL of media, and the pellet was mixed well into solution.

### 5.3 MTT assay

The first method used to test the *in vitro* cytotoxicity of our compounds was the MTT Assay. For our tests, we used 48-well plates. 1 mL of each cell line's respective media was placed into each well of the 48-well plates, and 1 drop of the final solution of the cells listed in the previous section were be added to each well and allowed to grow to 75 % confluency (covering 75% of the bottom of the well). Next, our products were added to the wells at a concentration of 100 μM, except for four wells in which DMSO was added so that the final concentration was 0.01% (negative control). Once the PBD products were added in triplicate, the plates were incubated for 48 hours. After 48 hours of incubation, 5-diphenyl-tetrazolium bromide (MTT) was added at 100 μg/well and will be incubated for 3–4 hours. After 3–4 hours of incubation, the supernatant fluid was removed and 0.1 M HCl in isopropanol was added to each well to dissolve the resulting formazan crystals. The crystals were dissolved into solution well using a transfer pipette. The optical density of the resulting solution was measured at 570 nm. The optical density of the solution of the formazan crystals is directly correlated to the remaining number of viable cells in solution. Percent cell viability was calculated by comparing the concentration of formazan crystals formed in the negative control to the formazan crystals formed by the sample in which our PBD products are added. The cell viability of the negative controls were taken to be 100%.

## Declarations

### Author contribution statement

Abbas Shilabin: Conceived and designed the experiments; Performed the experiments; Analyzed and interpreted the data; Wrote the paper.

Victoria Palau, John Jarrett, Crystal Whitted: Performed the experiments.

Joel Annor-Gyamfi, Joseph Osazee: Performed the experiments; Analyzed and interpreted the data.

Dobrusia Bialonska: Conceived and designed the experiments; Analyzed and interpreted the data; Wrote the paper.

### Funding statement

This work was supported by the ETSU Office of Research and Sponsored Programs Administration (ORSPA), RDC Major Grant (17-005M).

### Competing interest statement

The authors declare no conflict of interest.

### Additional information

No additional information is available for this paper.
